# Population attributable fractions for ovarian cancer in Swedish women by morphological type

**DOI:** 10.1038/sj.bjc.6604135

**Published:** 2007-12-11

**Authors:** C Granström, J Sundquist, K Hemminki

**Affiliations:** 1 Department of Neurobiology, Caring Sciences and Society, Center for Family and Community Medicine, Karolinska Institute, Alfred Nobels allé 12, Huddinge 141 83, Sweden; 2 Department of Medicine, Stanford Prevention Research Center, Stanford University School of Medicine, California, USA; 3 Division of Molecular Genetic Epidemiology, German Cancer Research Center (DKFZ), Heidelberg, Germany

**Keywords:** parity, family history, population-attributable fraction, relative risk, morphological types

## Abstract

Using the Swedish Family-Cancer Database, among a total of 1 030 806 women followed from 1993 through 2004, invasive and borderline epithelial ovarian cancer was identified in 3306 and 822 women respectively, with data on family history, reproductive variables, residential region and socioeconomic status. Relative risks and population-attributable fractions (PAFs) were estimated by Poisson regression. The overall PAFs of invasive epithelial ovarian cancer for family history and for reproductive factors were 2.6 and 22.3%, respectively, for serous/seropapillary cystadenocarcinoma (3.0 and 19.1%), endometrioid carcinoma (2.6 and 26.6%), mucinous cystadenocarcinoma (0.5 and 23.9%) and clear-cell carcinoma (2.6 and 73.9%). The corresponding PAFs of borderline tumours due to family history were lower, but higher due to reproductive factors. Family history, low parity and young age at first birth were associated with elevated risks. The risks for women with a family history were among the highest, but these women accounted for the smallest proportion of the cases, giving the lowest PAFs.

Most cases of invasive epithelial ovarian cancer (EOC), which constitute 80–90% of all ovarian malignancies, are detected at an advanced stage after age 40 years (Holschneider and Berek, 2000). Invasive EOC can be further subdivided into serous (75–80%), mucinous (10%), endometrioid (10%), clear-cell and other morphological types (Holschneider and Berek, 2000). Most studies suggest that apart from mucinous tumours, all the major morphologies tend to share a common aetiology (Whiteman *et al*, 2000; Purdie *et al*, 2003; Tung *et al*, 2003; Parazzini *et al*, 2004; Chiaffarino *et al*, 2007). There is strong evidence that multiparity and oral contraceptive use are protective factors (Adami *et al*, 1994; La Vecchia, 2006), as well as tubal ligation and hysterectomy (Green *et al*, 1997; Kjaer *et al*, 2004). Breastfeeding has shown a weaker inverse association, but not all studies have observed this (Riman *et al*, 2002; Chiaffarino *et al*, 2005). Additional risk factors include nulliparity and infertility (Banks *et al*, 1997), but the findings on the use of hormone replacement therapy are inconsistent (Riman *et al*, 2002; Kurian *et al*, 2005). Another well-established risk factor is a family history of EOC (Hemminki and Granstrom, 2003). The strongest association has been observed for the non-mucinous morphologies, whereas in one study the mucinous type was not associated with a family history (Purdie *et al*, 2001).

We use here the year 2006 update of the nation-wide Swedish Family-Cancer Database to determine relative risks (RRs) and population-attributable fractions (PAFs) of invasive and borderline EOC for reproductive, familial, socioeconomic and residential factors.

## MATERIALS AND METHODS

Statistics Sweden created a family database, ‘Second Generation Register’ in 1995. After a few expansions, it covered offspring born after 1931 with their parents, renamed ‘Multigeneration Register’, to indicate that the number of generations was more than two. We have linked this Register to the Swedish Cancer Registry (1958–2004) to make the Family-Cancer Database (MigMed2) in year 2006 for the seventh time. This now contains data on all immigrants, whereas previous versions only included those who had had children in Sweden. All data are organised in child–mother–father triplets; the parents have been registered at the time of birth of the child, allowing tracking of biological parents. The database includes all persons resident in Sweden after 1931 with their biological parents, totalling over 11.5 million individuals. The present study included Swedish-born as well as immigrant women born between years 1932 and 1953, that is, those whose minimal age at the beginning of the follow-up ranged from 40 to 61 years.

The completeness of cancer registration in the 1970s has been estimated to be over 95%, and is now considered close to 100%. The percentage of cytologically or histologically verified cases of EOC has been close to 100% (Center for Epidemiology, 2007). The Swedish Cancer Registry is based on compulsory notification of cases (Center for Epidemiology, 2007). A four-digit diagnostic code according to the International Classification of Diseases, seventh revision was combined with morphology codes according to the International Classification of Diseases for Oncology, World Health Organization (WHO) and the Systematized Nomenclature of Medicine (SNOMED) used since 1993. The ICD-7 code 175 was used to identify EOC cases, and a Swedish version of SNOMED was used to classify the morphological subtypes.

Follow-up was started at immigration or January 1, 1993, whichever came latest, and terminated on diagnosis of first cancer, death, emigration or the closing date of the study, December 31, 2004. A Poisson regression analysis was performed to model overall EOC incidence using the following variables: age at diagnosis (5-year bands), family history of invasive EOC, parity, age at first child birth, socioeconomic status and residential area (Clayton and Schifflers, 1987a, 1987b). A separate Poisson regression analysis was also performed for each morphology: serous, endometrioid, mucinous and clear cell. Epithelial borderline tumours were considered as a whole. Relative risks and confidence intervals (95% CIs) were calculated using Poisson regression. Although not shown in the results, age at diagnosis was always included in the model.

The PAF is the proportion of disease cases in a population that is attributable to a particular exposure or cause. Using the relevant variables from the Poisson regression models, individual PAFs due to each of the variables were calculated on the basis of RRs generated by new Poisson regression models with the variables classified dichotomously (exposed/unexposed). The population belonging to the lowest level of the variables in the initial Poisson regression models was defined as unexposed and the other levels were aggregated into the exposed group. The population-attributable fraction was calculated according to the formula ((RR−1)/RR) × the proportion of cases in the exposed population, where RR was the risk in the exposed population (Miettinen, 1974). For joint PAFs, defining the unexposed population as those unexposed to all risk factors simultaneously, we used the formula ((inc_all_−inc_ref_)/inc_all_) × 100, where inc_all_ denotes the overall incidence and inc_ref_ denotes that in the unexposed population. The incidence was age-standardised according to the Swedish census of year 2000. Confidence intervals for PAFs were estimated by bootstrapping with 1000 simulations (Greenland, 2004).

## RESULTS

A total of 1 030 806 women, with 11 091 139 person-years at risk were included, were followed from 1993 through 2004. Invasive and borderline EOC were identified in 3306 and 822 women, respectively, with data on family history, reproductive variables, socioeconomic status and residential region. Variables included in the Poisson regression models were age at diagnosis, family history, parity, age at first child birth, all of which had a significant or borderline significant effect on the risk of EOC; additionally, the effect of socioeconomic status and residential area were tested. The result of the Poisson analysis is shown in Table 1. A family history through a mother proband increased the risk of overall invasive EOC to 2.54, and through a sister to 2.76 relative to women with no family history. Nulliparity showed the highest RR, with an overall RR of 2.12 relative to those who had at least three children. Low parity increased RRs, as well as younger ages at first childbirth. There were small or no risks associated with residential region and socioeconomic status in the overall invasive EOC, but, for completeness, these variables were still included in the model although their exclusion did not alter risks associated with the other variables.

The invasive cases comprised 45% serous/seropapillary cystadeno, 15% endometrioid, 8% mucinous cystadeno and 5% clear-cell cystadenocarcinomas. Poisson regression analysis for these are being shown in Table 1. As in the overall EOC, these types showed positive associations with family history through a mother or sister proband (serous: 2.93 and 2.57, respectively; endometrioid: 2.22 and 3.65, respectively; clear cell: 2.63 and 2.40, respectively), except in mucinous cystadenocarcinoma of which there were very few cases. There was a clear trend for decreasing RRs with increasing parity in all morphologies, except mucinous tumours, which showed no trend. A similar trend for decreasing RRs with increasing age at first child birth was observed in the serous group, but with clear cell morphology this was reversed. No clear trend was observed for age at first child birth in the mucinous and endometrioid morphologies. Nulliparity showed the highest RRs in clear-cell carcinoma, with an overall significant RR of 6.73 relative to those who had at least three children, whereas serous/seropapillary and endometrioid cystadenocarcinomas had significant RRs of 1.86 and 2.77 respectively. In general, low parity had the highest impact on clear cell carcinoma. Unlike the other morphologies, mucinous cystadenocarcinoma was not positively associated with nulliparity. Residential region had significant or borderline significant moderately elevated risks, ranging from 1.11 to 1.35, with the reference groups north or south in all morphologies except from mucinous. The highest RRs were found in the clear cell morphology. There was a negative, but insignificant, association with high socioeconomic status and endometrioid, mucinous and clear-cell tumours as opposed to the serous morphology where the results were harder to interpret as all the defined socioeconomic groups had increased but very similar RRs.

The corresponding result for all epithelial borderline tumours is shown in Table 1. Here, family history refers to family history of invasive disease. The association with a mother history was weaker compared with the corresponding invasive analysis, but these RRs were not significant. A trend for decreasing RRs with increasing parity and also a similar trend for decreasing RRs with increasing age at first child birth was observed. Low parity showed higher RRs compared with the corresponding invasive model. Residential region had no elevated risk, but there was a negative association with high socioeconomic status.

For calculation of PAF, the individual variables in Table 1 were classified dichotomously into one group, the reference or unexposed group, and into the other, the exposed group (Table 2). As nulliparous women have no age at first birth, parity and age at first birth were merged into one variable. In order to calculate a joint PAF, all the unexposed groups of the individual variables were combined simultaneously to form the joint reference group. Socioeconomic status was excluded in this analysis due to the limited number of cases in the joint reference group, but this did not alter the other individual PAFs. The joint PAFs in all invasive EOC and mucinous carcinoma were similar, 26.5 and 29.6%, respectively, which was equal to the individual PAFs due to reproductive factors. Serous/seropapillary cystadeno and endometrioid carcinomas had large joint PAFs of 49.4 and 48.5%, respectively, explaining half of all cases. Clear-cell carcinoma showed a notable large PAF of 66.0%, most likely due to only one case in the joint reference group. The joint PAF in serous/seropapillary cystadeno and endometrioid carcinomas exceeded the sum of the individual PAFs, whereas in all EOC, mucinous cystadeno and clear-cell carcinomas the corresponding sum exceeded the joint PAF, perhaps because some risk factors cancel each other out. The corresponding joint PAF for the borderline tumours was equal.

## DISCUSSION

The major strength of this study is the large number of women from the nation-wide Swedish Family-Cancer Database and the registered and unbiased information on all variables. One limitation of the data source is the lack of information on known or potential risk/protective factors such as oral contraceptive use (Franceschi *et al*, 1991; Kumle *et al*, 2004), hormone replacement therapy (Mills *et al*, 2005; Danforth *et al*, 2007), breast feeding (Riman *et al*, 2002; Chiaffarino *et al*, 2005), menstrual history (Zografos *et al*, 2004; Riman *et al*, 2004b), dietary habits (Bosetti *et al*, 2001; Larsson *et al*, 2004) and obesity (Riman *et al*, 2004a; Olsen *et al*, 2007).

In this study family history of disease, low parity and young age at first birth increased the risk of both non-mucinous invasive and borderline EOC. Due to very few cases, no clear conclusions could be drawn regarding the risk of family history in the mucinous morphology. Low socioeconomic status seemed to be associated with borderline tumours as opposed to residential region.

Population-attributable fraction is the proportion of disease cases in a population that is attributable to a particular exposure or ‘the fraction of all cases (exposed and unexposed) that would not have occurred if exposure had not occurred (Miettinen, 1974; Rothman and Greenland, 1998; dos Santos Silva, 1999)’. Assuming exposure to be causal and removable, PAF could be used to estimate the potential impact of public health interventions. In this study however, exposures are not removable and PAF is used as an aetiological measure. The joint PAF of more than one exposure can analogously be defined as the fraction of cases that would not have occurred if all the exposures were avoided. Large PAFs indicate that a large proportion of the aetiology is understood at the level of the defined variable. Since PAF here is defined as the product of the expression ((RR−1)/RR) and the proportion of cases in the exposed population, large PAFs can only result from a high RR and relatively common exposure or from a moderate RR and common exposure.

No study has previously estimated morphology-specific PAFs for EOC, and few studies have estimated general PAFs (Parazzini *et al*, 2000; Hemminki and Granstrom, 2003). Our findings showed similar PAFs throughout all morphologies, with the exception of clear-cell carcinoma, regarding family history and reproductive factors. In these morphologies, family history accounted for the smallest PAFs (between 0.5 and 3.0%) due to the small proportion of exposed women, although these women had the highest RRs. A previous study estimated PAF due to family history of breast or ovarian cancer to 4% (Parazzini *et al*, 2000). The broad definition of exposure due to parity and age at first child birth resulted in very large (more than 80%) exposed groups, with resulting PAFs between 19.1 and 26.6%, although the corresponding RRs were low. An exception to this could be observed in clear-cell carcinoma where a high RR and an exceptionally small exposed group resulted in a reproduction-related PAF of 73.9%. The moderate to high PAFs due to being a resident in high-risk geographical areas seen in the specific morphologies are a possible result of potential risk/protective factors correlating with these areas, such as different use of oral contraceptives, hormone replacement therapy, obesity and dietary habits. These results could also be explained by different pathology classification criteria across medical regions. In two of the models, the serous/seropapillary and endometrioid morphologies, the sum of the individual PAFs was less than the corresponding joint PAF. According to these models, PAFs of 49.4 and 48.5% suggest that the three variables explain half of all cases. For specific morphological types, the interpretation is not clear as the residential region probably hides other factors contributing to the disease.

Also in the borderline model, family history accounted for the smallest individual PAF and reproductive factors for the highest PAFs. The borderline PAF was smaller than the corresponding invasive one due to family history, but larger due to reproductive factors, whereas the joint invasive and borderline PAFs were equal.

## Figures and Tables

**Table 1:** RRs in ovarian cancer according to the Poisson model

	**All epithelial**			**Serous/seropapillary cystadenocarcinoma**			**Endometrioid carcinoma**			**Mucinous cystadenocarcinoma**			**Clear-cell carcinoma**			**All epithelial borderline malignancy**		
**Variable**	**Cases**	**RR**	**(95% CI)**	**Cases**	**RR**	**(95% CI)**	**Cases**	**RR**	**(95% CI)**	**Cases**	**RR**	**(95% CI)**	**Cases**	**RR**	**(95% CI)**	**Cases**	**RR**	**(95% CI)**
*Family history*																		
Family history	140	2.60	(2.20–3.08)	68	2.83	(2.22–3.61)	21	2.61	(1.68–4.03)	6	1.32	(0.59–2.98)	8	2.57	(1.27–5.22)	21	1.51	(0.98–2.33)
Mother	99	2.54	(2.08–3.11)	51	2.93	(2.22–3.88)	13	2.22	(1.28–3.85)	3	0.92	(0.29–2.86)	6	2.63	(1.17–5.94)	12	1.18	(0.67–2.09)
Sister	41	2.76	(2.03–3.76)	17	2.57	(1.59–4.14)	8	3.65	(1.81–7.34)	3	2.39	(0.77–7.46)	2	2.40	(0.60–9.69)	9	2.38	(1.23–4.59)
No history	3166	1		1419	1		472	1		266	1		182	1		801	1	
																		
*Parity*																		
0	586	2.12	(1.81–2.48)	236	1.86	(1.47–2.37)	107	2.77	(1.85–4.16)	28	0.97	(0.54–1.72)	59	6.73	(3.38–13.42)	114	2.09	(1.50–2.92)
1	643	1.60	(1.43–1.79)	272	1.46	(1.24–1.73)	100	1.75	(1.31–2.33)	58	1.46	(1.01–2.10)	43	3.34	(1.97–5.67)	169	1.96	(1.57–2.45)
2	1329	1.21	(1.10–1.32)	630	1.23	(1.07–1.40)	180	1.16	(0.90–1.48)	113	1.05	(0.78–1.42)	65	1.85	(1.14–3.01)	371	1.49	(1.24–1.80)
3+	748	1		349	1		106	1		73	1		23	1		168	1	
																		
*Age at first birth*																		
13–20	637	1.13	(0.98–1.30)	293	1.17	(0.95–1.43)	99	1.31	(0.91–1.88)	58	1.03	(0.65–1.64)	25	1.03	(0.55–1.93)	176	1.48	(1.12–1.95)
21–24	925	1.08	(0.95–1.23)	444	1.15	(0.95–1.39)	119	1.04	(0.74–1.47)	79	0.96	(0.63–1.48)	40	1.04	(0.59–1.84)	246	1.30	(1.00–1.69)
25–29	814	1.06	(0.93–1.20)	361	1.02	(0.84–1.24)	119	1.15	(0.82–1.61)	75	1.05	(0.69–1.60)	47	1.28	(0.74–2.21)	205	1.18	(0.91–1.54)
30+	344	1		153	1		49	1		32	1		19	1		81	1	
																		
*Socioeconomic status*																		
Manual worker	1246	1.10	(1.01–1.19)	557	1.28	(1.06–1.56)	171	1.06	(0.78–1.42)	102	1.17	(0.77–1.78)	62	1.19	(0.72–1.95)	311	1.35	(1.05–1.73)
Professional	415	1.03	(0.92–1.15)	193	1.29	(1.03–1.62)	61	1		29	1		22	1		80	1	
Other	350	1.02	(0.90–1.15)	129	1		63	1.24	(0.87–1.77)	37	1.49	(0.91–2.43)	29	1.64	(0.94–2.86)	85	1.29	(0.95–1.75)
Blue collar	1295	1		608	1.26	(1.04–1.53)	198	1.02	(0.76–1.36)	104	1.09	(0.72–1.64)	77	1.11	(0.69–1.79)	346	1.30	(1.02–1.66)
																		
*Residential region*																		
Big city	1189	1.09	(0.99–1.20)	543	1.23	(1.06–1.42)	200	1.25	(1.02–1.53)	82	1		76	1.35	(0.89–2.05)	283	1	
South	1449	1.11	(1.01–1.21)	673	1.27	(1.10–1.46)	187	1		124	1.23	(0.93–1.63)	82	1.31	(0.87–1.97)	361	1.03	(0.88–1.21)
North	668	1		271	1		106	1.11	(0.88–1.41)	66	1.29	(0.93–1.79)	32	1		178	1.01	(0.83–1.21)
																		
All	3306			1487			493			272			190			822		

Abbreviations: CI=confidence interval: RR=relative risk.

Period of follow-up, 1993–2004.

**Table 2:** PAFs in EOC

	**Invasive**							**Borderline**					
**Morphology/variable**	**Cases**	**RR**	**(95% CI)**	**Prop (%)**	**PAF (%)**	**(95% CI)**	**Morphology/variable**	**Cases**	**RR**	**(95% CI)**	**Prop (%)**	**PAF (%)**	**(95% CI)**
*All epithelial invasive*							*All epithelial borderline malignancy*						
*Family history*							*Family history*						
Family history	140	2.60	(2.20–3.08)	4.23	2.6	(2.4–2.8)	Family history	21	1.51	(0.98–2.33)	2.55	0.9	(0.5–1.1)
No history	3166	1		95.77			No history	801	1		97.45		
													
*Parity and age at first birth*							*Parity and age at first birth*						
Other	2830	1.35	(1.23–1.49)	85.60	22.3	(20.3–24.0)	Other	729	1.76	(1.42–2.18)	88.69	38.3	(34.8–41.5)
3+: 21+	476	1		14.40			3+: 21+	93	1		11.31		
													
*Residential region*							*Residential region*						
Other	2638	1.10	(1.01–1.20)	79.79	7.2	(5.3–8.9)	Other	539	1.04	(0.90–1.20)	65.57	2.7	(0.3–5.3)
North	668	1		20.21			Big city	283	1		34.43		
													
*All*							*All*						
Other	3208			97.04	26.5	(22.0–34.5)	Other	787			95.74	24.8	(17.9–33.1)
All combined	98			2.96			All combined	35			4.26		
													
*Serous/seropapillary cystadenocarcinoma*													
*Family history*													
Family history	68	2.83	(2.22–3.61)	4.57	3.0	(2.6–3.2)							
No history	1419	1		95.43									
													
*Parity and age at first birth*													
Other	1263	1.29	(1.12–1.49)	84.94	19.1	(16.3–21.7)							
3+: 21+	224	1		15.06									
													
*Residential region*													
Other	1216	1.25	(1.10–1.43)	81.78	16.5	(13.9–19.3)							
North	271	1		18.22									
													
*All*													
Other	1454			97.78	49.4	(44.6–55.0)							
All combined	33			2.22									
													
*Endometrioid carcinoma*													
*Family history*													
Family history	21	2.61	(1.68–4.04)	4.26	2.6	(2.1–3.1)							
No history	472	1		95.74									
													
*Parity and age at first birth*													
Other	427	1.44	(1.11–1.87)	86.61	26.6	(21.8–31.3)							
3+: 21+	66	1		13.39									
													
*Residential region*													
Other	306	1.23	(1.02–1.47)	62.07	11.5	(8.7–14.3)							
North	187	1		37.93									
													
*All*													
Other	469			95.13	48.5	(43.0–55.3)							
All combined	24			4.87									
													
*Mucinous cystadenocarcinoma*													
*Family history*													
Family history	6	1.33	(0.59–2.98)	2.21	0.5	−(0.1–0.9)							
No history	266	1		97.79									
													
*Parity and age at first birth*													
Other	240	1.37	(0.95–1.98)	88.24	23.9	(17.7–31.3)							
2: 21–24	32	1		11.76									
													
*Residential region*													
Other	190	1.27	(0.98–1.64)	69.85	14.7	(10.4–18.9)							
Big city	82	1		30.15									
													
*All*													
Other	262			96.32	29.6	(20.3–45.7)							
All combined	10			3.68									
													
*Clear cell carcinoma*													
*Family history*													
Family history	8	2.57	(1.27–5.21)	4.21	2.6	(1.8–3.2)							
No history	182	1		95.79									
													
*Parity and age at first birth*													
Other	185	4.15	(1.71–10.09)	97.37	73.9	(70.2–82.4)							
3+: 13–20	5	1		2.63									
													
*Residential region*													
Other	158	1.35	(0.92–1.97)	83.16	21.3	(15.2–28.8)							
North	32	1		16.84									
													
*All*													
Other	189			99.47	66.0	(58.4–100)							
All combined	1			0.53									

Abbreviations: CI=confidence interval; EOC=epithelial ovarian cancer; PAF=population-attributable fraction; RR=relative risk.

Period of follow-up, 1993–2004.
